# 4-(2-Nitro­benzene­sulfonamido)pyridinium nitrate

**DOI:** 10.1107/S1600536808035654

**Published:** 2008-11-08

**Authors:** Liang Zhao, Qi-Fei Yu

**Affiliations:** a School of Food Science, Henan Institute of Science and Technology, Xinxiang 453003, People’s Republic of China; b Department of Food and Biological Engineering, Zhangzhou Institute of Technology, Henan University of Technology, Zhangzhou 363000, People’s Republic of China

## Abstract

There are two mol­ecules in the asymmetric unit of the title compound, C_11_H_10_N_3_O_4_S^+^·NO_3_^−^. All bond distances have normal values. The C—N bond distances in the sulfonamide group [1.389 (3) and 1.382 (3) Å] may indicate slight conjugation of the sulfonamide N-atom π-electrons with those of the pyridinium ring. The crystal structure is stabilized by N—H⋯O hydrogen bonds.

## Related literature

For zwitterionic forms of *N*–aryl­benzene­sulfonamides, see: Li *et al.* (2007 ▶); Yu & Li (2007 ▶). Damiano *et al.* (2007 ▶) describe the use of pyridinium derivatives for the construction of supra­molecular architectures. For bond-length data, see: Allen *et al.* (1987 ▶).
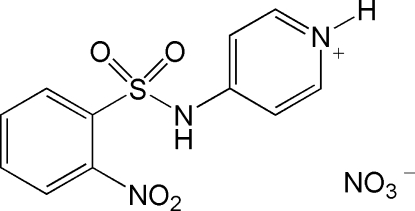


## Experimental

### 

#### Crystal data

C_11_H_10_N_3_O_4_S^+^·NO_3_^−^
*M* *_r_* = 342.30
Orthorhombic, 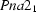

*a* = 14.716 (3) Å
*b* = 8.6671 (17) Å
*c* = 21.941 (4) Å
*V* = 2798.5 (9) Å^3^
*Z* = 8
Mo *K*α radiation
μ = 0.28 mm^−1^
*T* = 113 (2) K
0.20 × 0.16 × 0.02 mm

#### Data collection

Rigaku Saturn CCD area-detector diffractometer
Absorption correction: multi-scan (*CrystalClear*; Rigaku/MSC, 2005 ▶) *T*_min_ = 0.943, *T*_max_ = 0.998
20607 measured reflections
5734 independent reflections
5237 reflections with *I* > 2σ(*I*)
*R*_int_ = 0.045

#### Refinement

*R*[*F* ^2^ > 2σ(*F* ^2^)] = 0.040
*wR*(*F* ^2^) = 0.098
*S* = 1.04
5734 reflections
432 parameters
1 restraint
H atoms treated by a mixture of independent and constrained refinement
Δρ_max_ = 0.42 e Å^−3^
Δρ_min_ = −0.33 e Å^−3^
Absolute structure: Flack (1983 ▶), 2570 Friedel pairs
Flack parameter: 0.14 (6)

### 

Data collection: *CrystalClear* (Rigaku/MSC, 2005 ▶); cell refinement: *CrystalClear*; data reduction: *CrystalStructure* (Rigaku/MSC, 2005 ▶); program(s) used to solve structure: *SHELXS97* (Sheldrick, 2008 ▶); program(s) used to refine structure: *SHELXL97* (Sheldrick, 2008 ▶); molecular graphics: *SHELXTL* (Sheldrick, 2008 ▶); software used to prepare material for publication: *SHELXTL*.

## Supplementary Material

Crystal structure: contains datablocks global, I. DOI: 10.1107/S1600536808035654/rk2115sup1.cif

Structure factors: contains datablocks I. DOI: 10.1107/S1600536808035654/rk2115Isup2.hkl

Additional supplementary materials:  crystallographic information; 3D view; checkCIF report

## Figures and Tables

**Table 1:** Hydrogen-bond geometry (Å, °)

*D*—H⋯*A*	*D*—H	H⋯*A*	*D*⋯*A*	*D*—H⋯*A*
N1—H1*A*⋯O11^i^	1.06 (4)	1.68 (4)	2.745 (3)	179 (3)
N1—H1*A*⋯O9^i^	1.06 (4)	2.65 (3)	3.361 (3)	124 (2)
N4—H4*A*⋯O13^ii^	0.90 (3)	1.85 (3)	2.737 (3)	171 (2)
N4—H4*A*⋯O12^ii^	0.90 (3)	2.68 (3)	3.273 (3)	124 (2)
N5—H5*A*⋯O13^iii^	0.94 (3)	1.87 (3)	2.751 (3)	155 (2)
N2—H2*A*⋯O11^iv^	0.97 (4)	1.78 (4)	2.725 (3)	166 (4)

Symmetry codes: (i) 
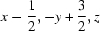
; (ii) 
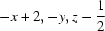
; (iii) 
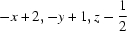
; (iv) 
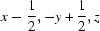
.

**Fig. 1.**
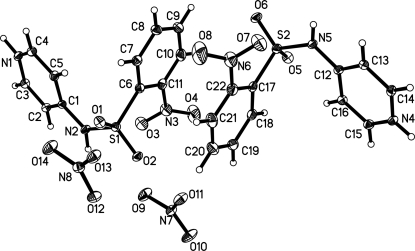
The molecular structure of the title compound, showing the two symmetry-independent molecules with the atom numbering scheme. Displacement ellipsoids are drawn at the 35% probability level. H atoms are presented as a small spheres of arbitrary radius.

## supplementary crystallographic information

### Comment

Organic pyridinium salts have been widely used in the construction of supramolecular architectures (Damiano *et al.*, 2007). As part of our ongoing studies of supramolecular chemistry involving the pyridinium rings (Li *et al.*, 2007), the structure of the title compound was determined by X–ray diffraction. In the cations of the title compound the short C–N distance [N2–C1 = 1.389 (3)Å and N5–C12 = 1.382 (3)Å] has a value between those of a typical C═N double and C—N single bond (1.47–1.50Å and 1.34–1.38Å, respectively; Allen *et al.*, 1987). This might be indicative of a slight conjugation of the N sulfonamide π–electrons with those of the pyridinium ring. In the two symmetry–independent molecules (Fig. 1), the dihedral angles between the benzene ring and the pyridinium ring are 85.1 (1)° and 86.2 (1)° respectively. The dihedral angles between the nitro–group and the benzene ring are 41.2 (1)° and 40.5 (2)° respectively.

### Experimental

A solution of 2–nitrobenzenesulfonyl chloride (2.2 g, 10 mmol) in CH_2_Cl_2_ (10 ml) was added dropwise to a suspension of 4–aminopyridine (0.9 g, 10 mmol) in CH_2_Cl_2_ (10 ml) at room temperature with stirring. The reaction mixture was stirred overnight. The yellow solid obtained was washed with warm water to obtain the title compound in a yield of 52.9%. A colourless single crystals, suitable for X–ray analysis were obtained by slow evaporation of an nitric acid (10%) solution at room temperature over a period of a week.

### Refinement

The N–bound H atoms were located in a difference map and refined isotropically. The C–bound H atoms were positioned geometrically (C—H = 0.95 Å) and refined as riding with *U*_iso_(H) = 1.2*U*_eq_(C).

### Crystal data

**Table:**

C_11_H_10_N_3_O_4_S^+^·NO_3_^–^	*F*_000_ = 1408
*M**_r_* = 342.30	*D*_x_ = 1.625 Mg m^−^^3^
Orthorhombic, *P**n**a*2_1_	Mo *K*α radiation λ = 0.71073 Å
Hall symbol: P 2c -2n	Cell parameters from 8078 reflections
*a* = 14.716 (3) Å	θ = 3.1–27.1º
*b* = 8.6671 (17) Å	µ = 0.28 mm^−^^1^
*c* = 21.941 (4) Å	*T* = 113 (2) K
*V* = 2798.5 (9) Å^3^	Block, colourless
*Z* = 8	0.20 × 0.16 × 0.02 mm

### Data collection

**Table:**

Rigaku Saturn CCD area-detector diffractometer	5734 independent reflections
Radiation source: Rotating anode	5237 reflections with *I* > 2σ(*I*)
Monochromator: Confocal	*R*_int_ = 0.046
Detector resolution: 7.31 pixels mm^-1^	θ_max_ = 27.1º
*T* = 113(2) K	θ_min_ = 3.3º
ω scans	*h* = −18→18
Absorption correction: multi-scan(CrystalClear; Rigaku/MSC, 2005)	*k* = −11→9
*T*_min_ = 0.943, *T*_max_ = 0.998	*l* = −20→28
20607 measured reflections	

### Refinement

**Table:**

Refinement on *F*^2^	Hydrogen site location: inferred from neighbouring sites
Least-squares matrix: Full	H atoms treated by a mixture of independent and constrained refinement
*R*[*F*^2^ > 2σ(*F*^2^)] = 0.040	*w* = 1/[σ^2^(*F*_o_^2^) + (0.0538*P*)^2^ + 0.0901*P*] where *P* = (*F*_o_^2^ + 2*F*_c_^2^)/3
*wR*(*F*^2^) = 0.098	(Δ/σ)_max_ = 0.001
*S* = 1.04	Δρ_max_ = 0.42 e Å^−^^3^
5734 reflections	Δρ_min_ = −0.33 e Å^−^^3^
432 parameters	Extinction correction: SHELXL97 (Sheldrick, 2008), Fc^*^=kFc[1+0.001xFc^2^λ^3^/sin(2θ)]^-1/4^
1 restraint	Extinction coefficient: 0.0054 (7)
Primary atom site location: structure-invariant direct methods	Absolute structure: Flack (1983), 2570 Friedel pairs
Secondary atom site location: difference Fourier map	Flack parameter: 0.14 (6)

### Special details

**Table:**

Geometry. All s.u.'s (except the s.u. in the dihedral angle between two l.s. planes) are estimated using the full covariance matrix. The cell s.u.'s are taken into account individually in the estimation of s.u.'s in distances, angles and torsion angles; correlations between s.u.'s in cell parameters are only used when they are defined by crystal symmetry. An approximate (isotropic) treatment of cell s.u.'s is used for estimating s.u.'s involving l.s. planes.
Refinement. Refinement of *F*^2^ against ALL reflections. The weighted *R*–factor *wR* and goodness of fit *S* are based on *F*^2^, conventional *R*–factors *R* are based on *F*, with *F* set to zero for negative *F*^2^. The threshold expression of *F*^2^ > σ(*F*^2^) is used only for calculating *R*–factors(gt) *etc*. and is not relevant to the choice of reflections for refinement. *R*–factors based on *F*^2^ are statistically about twice as large as those based on *F*, and *R*–factors based on ALL data will be even larger.

### Fractional atomic coordinates and isotropic or equivalent isotropic displacement parameters (Å^2^)

**Table:**

	*x*	*y*	*z*	*U*_iso_*/*U*_eq_	
S1	0.55489 (4)	0.54735 (7)	0.70897 (3)	0.01874 (14)	
S2	0.74559 (4)	0.43842 (7)	0.42871 (3)	0.02018 (14)	
O1	0.63055 (12)	0.5867 (2)	0.74667 (8)	0.0233 (4)	
O2	0.53170 (13)	0.3890 (2)	0.69999 (8)	0.0248 (4)	
O3	0.38958 (15)	0.5302 (3)	0.62281 (9)	0.0384 (5)	
O4	0.4342 (2)	0.4321 (4)	0.53747 (12)	0.0689 (10)	
O5	0.66949 (13)	0.3918 (2)	0.39273 (8)	0.0256 (4)	
O6	0.76670 (13)	0.5983 (2)	0.43544 (9)	0.0270 (4)	
O7	0.91479 (15)	0.4690 (3)	0.51112 (10)	0.0437 (6)	
O8	0.8745 (2)	0.5660 (4)	0.59706 (13)	0.0769 (11)	
N1	0.40728 (15)	1.0904 (3)	0.75443 (9)	0.0202 (4)	
N2	0.46295 (15)	0.6264 (2)	0.73697 (9)	0.0185 (4)	
N3	0.44496 (18)	0.5160 (3)	0.58163 (11)	0.0330 (6)	
N4	0.89765 (15)	−0.1009 (3)	0.37907 (9)	0.0186 (4)	
N5	0.83710 (16)	0.3598 (3)	0.40056 (9)	0.0200 (5)	
N6	0.86294 (19)	0.4797 (3)	0.55345 (11)	0.0361 (6)	
C1	0.44743 (16)	0.7834 (3)	0.74440 (10)	0.0160 (5)	
C2	0.35733 (17)	0.8310 (3)	0.75337 (10)	0.0173 (5)	
H2	0.3096	0.7576	0.7560	0.021*	
C3	0.33974 (17)	0.9853 (3)	0.75823 (11)	0.0191 (5)	
H3	0.2790	1.0192	0.7644	0.023*	
C4	0.49413 (18)	1.0467 (3)	0.74780 (11)	0.0199 (5)	
H4	0.5406	1.1227	0.7465	0.024*	
C5	0.51674 (19)	0.8933 (3)	0.74283 (11)	0.0205 (5)	
H5	0.5784	0.8626	0.7384	0.025*	
C6	0.57669 (18)	0.6366 (3)	0.63708 (11)	0.0206 (5)	
C7	0.65379 (17)	0.7288 (3)	0.63400 (11)	0.0220 (5)	
H7	0.6886	0.7462	0.6698	0.026*	
C8	0.6808 (2)	0.7962 (3)	0.57937 (12)	0.0255 (6)	
H8	0.7331	0.8602	0.5781	0.031*	
C9	0.6311 (2)	0.7697 (3)	0.52707 (12)	0.0279 (6)	
H9	0.6491	0.8159	0.4897	0.033*	
C10	0.5556 (2)	0.6764 (4)	0.52898 (12)	0.0307 (7)	
H10	0.5221	0.6572	0.4928	0.037*	
C11	0.52818 (19)	0.6104 (3)	0.58340 (12)	0.0226 (5)	
C12	0.85468 (17)	0.2042 (3)	0.39336 (10)	0.0180 (5)	
C13	0.94527 (17)	0.1584 (3)	0.38431 (10)	0.0181 (5)	
H13	0.9925	0.2331	0.3832	0.022*	
C14	0.96478 (19)	0.0056 (3)	0.37715 (11)	0.0206 (5)	
H14	1.0258	−0.0262	0.3708	0.025*	
C15	0.80967 (19)	−0.0599 (3)	0.38700 (11)	0.0214 (5)	
H15	0.7638	−0.1370	0.3875	0.026*	
C16	0.78660 (18)	0.0911 (3)	0.39421 (11)	0.0190 (5)	
H16	0.7248	0.1196	0.3998	0.023*	
C17	0.72784 (18)	0.3544 (3)	0.50237 (11)	0.0207 (5)	
C18	0.65166 (18)	0.2619 (3)	0.50870 (11)	0.0247 (6)	
H18	0.6141	0.2427	0.4743	0.030*	
C19	0.6294 (2)	0.1970 (3)	0.56452 (12)	0.0283 (6)	
H19	0.5777	0.1320	0.5678	0.034*	
C20	0.6817 (2)	0.2263 (4)	0.61506 (13)	0.0336 (7)	
H20	0.6664	0.1814	0.6532	0.040*	
C21	0.7570 (2)	0.3217 (4)	0.61016 (13)	0.0337 (7)	
H21	0.7926	0.3443	0.6452	0.040*	
C22	0.7801 (2)	0.3839 (3)	0.55443 (12)	0.0275 (6)	
O9	0.71160 (13)	0.2133 (2)	0.76947 (9)	0.0310 (5)	
O10	0.72427 (15)	−0.0296 (2)	0.74762 (11)	0.0348 (5)	
O11	0.84491 (13)	0.1111 (2)	0.75566 (10)	0.0280 (4)	
N7	0.75865 (15)	0.0975 (2)	0.75795 (10)	0.0198 (5)	
O12	0.91094 (12)	0.2926 (2)	0.88498 (8)	0.0256 (4)	
O13	1.04400 (13)	0.4007 (2)	0.88032 (9)	0.0257 (4)	
O14	0.92515 (15)	0.5367 (2)	0.90341 (10)	0.0318 (5)	
N8	0.95891 (15)	0.4105 (2)	0.89013 (9)	0.0193 (4)	
H1A	0.384 (2)	1.207 (4)	0.7547 (15)	0.044 (9)*	
H4A	0.9161 (19)	−0.199 (4)	0.3751 (12)	0.017 (7)*	
H5A	0.890 (2)	0.420 (3)	0.3983 (12)	0.016 (7)*	
H2A	0.413 (3)	0.554 (5)	0.7422 (17)	0.053 (11)*	

### Atomic displacement parameters (Å^2^)

**Table:**

	*U*^11^	*U*^22^	*U*^33^	*U*^12^	*U*^13^	*U*^23^
S1	0.0182 (3)	0.0171 (3)	0.0209 (3)	0.0031 (2)	0.0009 (2)	0.0013 (2)
S2	0.0189 (3)	0.0176 (3)	0.0240 (3)	0.0032 (2)	0.0005 (2)	0.0011 (2)
O1	0.0181 (10)	0.0296 (11)	0.0220 (9)	0.0047 (8)	−0.0031 (7)	0.0024 (7)
O2	0.0272 (11)	0.0151 (9)	0.0320 (10)	0.0040 (8)	0.0058 (8)	0.0004 (7)
O3	0.0316 (12)	0.0502 (15)	0.0333 (11)	−0.0108 (11)	−0.0023 (9)	−0.0030 (9)
O4	0.070 (2)	0.090 (2)	0.0469 (15)	−0.0440 (18)	0.0051 (13)	−0.0351 (14)
O5	0.0189 (10)	0.0325 (12)	0.0254 (9)	0.0035 (8)	−0.0028 (7)	0.0023 (7)
O6	0.0298 (11)	0.0140 (9)	0.0371 (11)	0.0046 (7)	0.0045 (9)	0.0017 (7)
O7	0.0301 (13)	0.0669 (18)	0.0341 (12)	−0.0137 (11)	−0.0006 (9)	−0.0057 (10)
O8	0.070 (2)	0.101 (3)	0.0595 (18)	−0.0401 (19)	0.0026 (15)	−0.0462 (16)
N1	0.0251 (12)	0.0147 (11)	0.0208 (10)	0.0011 (9)	−0.0024 (9)	−0.0011 (8)
N2	0.0158 (11)	0.0135 (11)	0.0262 (10)	0.0016 (8)	0.0016 (8)	−0.0003 (8)
N3	0.0296 (14)	0.0409 (16)	0.0284 (12)	−0.0082 (12)	−0.0055 (10)	−0.0036 (11)
N4	0.0179 (11)	0.0147 (12)	0.0231 (10)	0.0026 (9)	−0.0016 (8)	0.0009 (7)
N5	0.0192 (12)	0.0144 (11)	0.0263 (11)	0.0003 (9)	0.0039 (8)	−0.0004 (8)
N6	0.0300 (15)	0.0478 (17)	0.0305 (13)	−0.0070 (13)	−0.0056 (11)	−0.0082 (11)
C1	0.0168 (12)	0.0138 (12)	0.0175 (11)	0.0007 (9)	0.0015 (9)	0.0000 (8)
C2	0.0146 (12)	0.0166 (12)	0.0207 (11)	−0.0010 (10)	−0.0031 (9)	0.0004 (9)
C3	0.0134 (12)	0.0205 (13)	0.0233 (12)	0.0026 (10)	0.0010 (9)	−0.0004 (9)
C4	0.0188 (13)	0.0196 (13)	0.0215 (11)	−0.0007 (10)	−0.0011 (10)	0.0000 (9)
C5	0.0193 (14)	0.0206 (14)	0.0216 (12)	0.0004 (10)	−0.0001 (10)	−0.0018 (9)
C6	0.0236 (13)	0.0184 (13)	0.0197 (11)	0.0041 (10)	−0.0008 (9)	0.0007 (9)
C7	0.0207 (14)	0.0209 (14)	0.0243 (12)	0.0011 (11)	−0.0001 (10)	−0.0006 (10)
C8	0.0233 (14)	0.0241 (14)	0.0290 (13)	−0.0010 (11)	0.0055 (11)	0.0020 (10)
C9	0.0329 (16)	0.0268 (15)	0.0239 (12)	0.0025 (12)	0.0048 (11)	0.0027 (10)
C10	0.0366 (18)	0.0333 (17)	0.0223 (14)	0.0011 (13)	−0.0035 (11)	−0.0054 (11)
C11	0.0202 (15)	0.0224 (14)	0.0252 (12)	−0.0001 (11)	−0.0015 (10)	−0.0053 (9)
C12	0.0236 (13)	0.0152 (12)	0.0150 (10)	0.0008 (10)	−0.0038 (9)	0.0017 (8)
C13	0.0179 (12)	0.0184 (13)	0.0180 (11)	−0.0005 (10)	0.0003 (9)	0.0004 (9)
C14	0.0236 (14)	0.0196 (13)	0.0187 (11)	0.0019 (11)	−0.0011 (9)	−0.0027 (9)
C15	0.0218 (14)	0.0196 (13)	0.0229 (12)	−0.0038 (10)	−0.0004 (10)	0.0000 (9)
C16	0.0159 (12)	0.0194 (13)	0.0217 (11)	−0.0005 (10)	−0.0002 (10)	0.0001 (9)
C17	0.0227 (13)	0.0173 (13)	0.0220 (12)	0.0044 (11)	0.0008 (9)	−0.0030 (9)
C18	0.0249 (14)	0.0207 (14)	0.0284 (13)	0.0010 (11)	0.0029 (11)	−0.0039 (10)
C19	0.0270 (15)	0.0246 (15)	0.0334 (14)	0.0008 (12)	0.0072 (11)	0.0002 (11)
C20	0.0361 (18)	0.0377 (19)	0.0270 (13)	0.0117 (14)	0.0054 (12)	0.0057 (11)
C21	0.0359 (18)	0.0425 (19)	0.0228 (13)	0.0043 (14)	−0.0046 (11)	−0.0037 (11)
C22	0.0276 (17)	0.0276 (15)	0.0273 (13)	−0.0009 (12)	0.0002 (11)	−0.0040 (10)
O9	0.0206 (10)	0.0199 (11)	0.0526 (12)	0.0062 (8)	0.0013 (9)	−0.0032 (8)
O10	0.0240 (11)	0.0164 (10)	0.0641 (14)	−0.0049 (8)	−0.0049 (10)	−0.0047 (9)
O11	0.0155 (10)	0.0191 (10)	0.0495 (12)	−0.0017 (8)	0.0039 (8)	−0.0025 (8)
N7	0.0179 (12)	0.0159 (11)	0.0255 (11)	−0.0003 (9)	−0.0007 (8)	−0.0014 (8)
O12	0.0231 (10)	0.0196 (10)	0.0341 (10)	−0.0052 (8)	−0.0010 (8)	0.0019 (7)
O13	0.0191 (10)	0.0188 (10)	0.0392 (11)	0.0021 (7)	0.0008 (8)	−0.0024 (8)
O14	0.0245 (10)	0.0203 (11)	0.0505 (13)	0.0047 (8)	0.0014 (9)	−0.0046 (8)
N8	0.0191 (11)	0.0188 (11)	0.0200 (10)	0.0003 (9)	−0.0028 (8)	0.0021 (8)

### Geometric parameters (Å, °)

**Table:**

S1—O2	1.4281 (19)	C6—C11	1.396 (4)
S1—O1	1.4284 (19)	C7—C8	1.391 (4)
S1—N2	1.636 (2)	C7—H7	0.9500
S1—C6	1.786 (2)	C8—C9	1.379 (4)
S2—O6	1.4275 (19)	C8—H8	0.9500
S2—O5	1.429 (2)	C9—C10	1.375 (4)
S2—N5	1.631 (2)	C9—H9	0.9500
S2—C17	1.792 (3)	C10—C11	1.385 (4)
O3—N3	1.223 (3)	C10—H10	0.9500
O4—N3	1.222 (3)	C12—C16	1.402 (4)
O7—N6	1.206 (3)	C12—C13	1.405 (4)
O8—N6	1.226 (4)	C13—C14	1.365 (4)
N1—C4	1.341 (3)	C13—H13	0.9500
N1—C3	1.351 (3)	C14—H14	0.9500
N1—H1A	1.06 (4)	C15—C16	1.361 (4)
N2—C1	1.389 (3)	C15—H15	0.9500
N2—H2A	0.97 (4)	C16—H16	0.9500
N3—C11	1.473 (4)	C17—C18	1.385 (4)
N4—C14	1.352 (4)	C17—C22	1.400 (4)
N4—C15	1.354 (3)	C18—C19	1.387 (4)
N4—H4A	0.90 (3)	C18—H18	0.9500
N5—C12	1.382 (3)	C19—C20	1.374 (4)
N5—H5A	0.94 (3)	C19—H19	0.9500
N6—C22	1.476 (4)	C20—C21	1.387 (4)
C1—C5	1.396 (3)	C20—H20	0.9500
C1—C2	1.403 (3)	C21—C22	1.379 (4)
C2—C3	1.366 (4)	C21—H21	0.9500
C2—H2	0.9500	O9—N7	1.245 (3)
C3—H3	0.9500	O10—N7	1.233 (3)
C4—C5	1.375 (3)	O11—N7	1.276 (3)
C4—H4	0.9500	O12—N8	1.248 (3)
C5—H5	0.9500	O13—N8	1.273 (3)
C6—C7	1.390 (4)	O14—N8	1.236 (3)
			
O2—S1—O1	119.72 (12)	C9—C8—C7	119.7 (3)
O2—S1—N2	104.89 (12)	C9—C8—H8	120.1
O1—S1—N2	109.10 (11)	C7—C8—H8	120.1
O2—S1—C6	109.71 (12)	C10—C9—C8	120.0 (2)
O1—S1—C6	105.54 (12)	C10—C9—H9	120.0
N2—S1—C6	107.38 (12)	C8—C9—H9	120.0
O6—S2—O5	120.17 (12)	C9—C10—C11	120.4 (3)
O6—S2—N5	105.37 (12)	C9—C10—H10	119.8
O5—S2—N5	108.65 (12)	C11—C10—H10	119.8
O6—S2—C17	109.44 (12)	C10—C11—C6	120.7 (3)
O5—S2—C17	105.61 (12)	C10—C11—N3	116.7 (2)
N5—S2—C17	106.99 (12)	C6—C11—N3	122.5 (2)
C4—N1—C3	121.1 (2)	N5—C12—C16	123.2 (2)
C4—N1—H1A	125.2 (19)	N5—C12—C13	118.0 (2)
C3—N1—H1A	113.6 (19)	C16—C12—C13	118.8 (2)
C1—N2—S1	126.18 (18)	C14—C13—C12	119.4 (2)
C1—N2—H2A	120 (2)	C14—C13—H13	120.3
S1—N2—H2A	113 (2)	C12—C13—H13	120.3
O4—N3—O3	124.0 (3)	N4—C14—C13	120.3 (2)
O4—N3—C11	117.3 (3)	N4—C14—H14	119.8
O3—N3—C11	118.6 (2)	C13—C14—H14	119.8
C14—N4—C15	121.6 (2)	N4—C15—C16	120.4 (2)
C14—N4—H4A	115.1 (18)	N4—C15—H15	119.8
C15—N4—H4A	123.4 (18)	C16—C15—H15	119.8
C12—N5—S2	127.28 (19)	C15—C16—C12	119.5 (2)
C12—N5—H5A	112.8 (17)	C15—C16—H16	120.2
S2—N5—H5A	117.9 (17)	C12—C16—H16	120.2
O7—N6—O8	124.1 (3)	C18—C17—C22	117.9 (2)
O7—N6—C22	119.4 (2)	C18—C17—S2	116.33 (18)
O8—N6—C22	116.5 (3)	C22—C17—S2	125.6 (2)
N2—C1—C5	123.0 (2)	C17—C18—C19	121.0 (2)
N2—C1—C2	117.4 (2)	C17—C18—H18	119.5
C5—C1—C2	119.6 (2)	C19—C18—H18	119.5
C3—C2—C1	118.6 (2)	C20—C19—C18	120.4 (3)
C3—C2—H2	120.7	C20—C19—H19	119.8
C1—C2—H2	120.7	C18—C19—H19	119.8
N1—C3—C2	121.1 (2)	C19—C20—C21	119.7 (3)
N1—C3—H3	119.5	C19—C20—H20	120.2
C2—C3—H3	119.5	C21—C20—H20	120.2
N1—C4—C5	120.8 (2)	C22—C21—C20	119.9 (3)
N1—C4—H4	119.6	C22—C21—H21	120.0
C5—C4—H4	119.6	C20—C21—H21	120.0
C4—C5—C1	118.7 (2)	C21—C22—C17	121.1 (3)
C4—C5—H5	120.6	C21—C22—N6	115.9 (3)
C1—C5—H5	120.6	C17—C22—N6	123.0 (2)
C7—C6—C11	118.0 (2)	O10—N7—O9	121.9 (2)
C7—C6—S1	116.03 (18)	O10—N7—O11	118.9 (2)
C11—C6—S1	125.7 (2)	O9—N7—O11	119.1 (2)
C6—C7—C8	121.1 (2)	O14—N8—O12	121.3 (2)
C6—C7—H7	119.5	O14—N8—O13	119.6 (2)
C8—C7—H7	119.5	O12—N8—O13	119.1 (2)
			
O2—S1—N2—C1	−169.3 (2)	O3—N3—C11—C10	−137.1 (3)
O1—S1—N2—C1	61.3 (2)	O4—N3—C11—C6	−141.4 (3)
C6—S1—N2—C1	−52.7 (2)	O3—N3—C11—C6	41.3 (4)
O6—S2—N5—C12	170.8 (2)	S2—N5—C12—C16	17.8 (3)
O5—S2—N5—C12	−59.2 (2)	S2—N5—C12—C13	−162.69 (19)
C17—S2—N5—C12	54.4 (2)	N5—C12—C13—C14	179.9 (2)
S1—N2—C1—C5	−16.3 (3)	C16—C12—C13—C14	−0.6 (3)
S1—N2—C1—C2	163.17 (18)	C15—N4—C14—C13	1.2 (3)
N2—C1—C2—C3	−177.3 (2)	C12—C13—C14—N4	−0.4 (3)
C5—C1—C2—C3	2.2 (3)	C14—N4—C15—C16	−1.0 (4)
C4—N1—C3—C2	−2.3 (4)	N4—C15—C16—C12	0.0 (3)
C1—C2—C3—N1	0.1 (3)	N5—C12—C16—C15	−179.8 (2)
C3—N1—C4—C5	2.0 (4)	C13—C12—C16—C15	0.8 (3)
N1—C4—C5—C1	0.4 (3)	O6—S2—C17—C18	133.3 (2)
N2—C1—C5—C4	177.0 (2)	O5—S2—C17—C18	2.6 (2)
C2—C1—C5—C4	−2.5 (3)	N5—S2—C17—C18	−113.0 (2)
O2—S1—C6—C7	−134.5 (2)	O6—S2—C17—C22	−41.5 (3)
O1—S1—C6—C7	−4.3 (2)	O5—S2—C17—C22	−172.2 (2)
N2—S1—C6—C7	112.0 (2)	N5—S2—C17—C22	72.2 (3)
O2—S1—C6—C11	39.3 (3)	C22—C17—C18—C19	−1.8 (4)
O1—S1—C6—C11	169.6 (2)	S2—C17—C18—C19	−177.1 (2)
N2—S1—C6—C11	−74.1 (3)	C17—C18—C19—C20	1.5 (4)
C11—C6—C7—C8	1.5 (4)	C18—C19—C20—C21	0.2 (4)
S1—C6—C7—C8	175.8 (2)	C19—C20—C21—C22	−1.5 (5)
C6—C7—C8—C9	−0.9 (4)	C20—C21—C22—C17	1.0 (5)
C7—C8—C9—C10	−0.3 (4)	C20—C21—C22—N6	−178.1 (3)
C8—C9—C10—C11	0.9 (4)	C18—C17—C22—C21	0.6 (4)
C9—C10—C11—C6	−0.3 (4)	S2—C17—C22—C21	175.3 (2)
C9—C10—C11—N3	178.1 (3)	C18—C17—C22—N6	179.6 (3)
C7—C6—C11—C10	−0.9 (4)	S2—C17—C22—N6	−5.6 (4)
S1—C6—C11—C10	−174.7 (2)	O7—N6—C22—C21	139.4 (3)
C7—C6—C11—N3	−179.2 (3)	O8—N6—C22—C21	−40.2 (4)
S1—C6—C11—N3	7.0 (4)	O7—N6—C22—C17	−39.7 (4)
O4—N3—C11—C10	40.2 (4)	O8—N6—C22—C17	140.7 (3)

### Hydrogen-bond geometry (Å, °)

**Table:**

*D*—H···*A*	*D*—H	H···*A*	*D*···*A*	*D*—H···*A*
N1—H1A···O11^i^	1.06 (4)	1.68 (4)	2.745 (3)	179 (3)
N1—H1A···O9^i^	1.06 (4)	2.65 (3)	3.361 (3)	124 (2)
N4—H4A···O13^ii^	0.90 (3)	1.85 (3)	2.737 (3)	171 (2)
N4—H4A···O12^ii^	0.90 (3)	2.68 (3)	3.273 (3)	124 (2)
N5—H5A···O13^iii^	0.94 (3)	1.87 (3)	2.751 (3)	155 (2)
N2—H2A···O11^iv^	0.97 (4)	1.78 (4)	2.725 (3)	166 (4)

Symmetry codes: (i) *x*−1/2, −*y*+3/2, *z*; (ii) −*x*+2, −*y*, *z*−1/2; (iii) −*x*+2, −*y*+1, *z*−1/2; (iv) *x*−1/2, −*y*+1/2, *z*.
